# Predictors of adherence to COVID-19 prevention measure among communities in North Shoa Zone, Ethiopia based on health belief model: A cross-sectional study

**DOI:** 10.1371/journal.pone.0246006

**Published:** 2021-01-22

**Authors:** Sisay Shewasinad Yehualashet, Kokebe Kefelegn Asefa, Alemayehu Gonie Mekonnen, Belete Negess Gemeda, Wondimenh Shibabaw Shiferaw, Yared Asmare Aynalem, Awraris Hailu Bilchut, Behailu Tariku Derseh, Abinet Dagnaw Mekuria, Wassie Negash Mekonnen, Wondesen Asegidew Meseret, Sisay Shine Tegegnework, Akine Eshete Abosetegn

**Affiliations:** 1 Department of Nursing, College of Health Science, Debre Berhan University, Debre Berhan, Amhara Regional State, Ethiopia; 2 Department of Public health, College of Health Science, Debre Berhan University, Debre Berhan, Amhara Regional State, Ethiopia; Ministry of Health and Sports, Myanmar, MYANMAR

## Abstract

**Introduction:**

Coronavirus disease 2019 (COVID-19) is an emerging respiratory infections and is known to cause illness ranging from the common cold to severe acute respiratory syndrome. At present, the disease has been posing a serious threat to the communities, and it is critical to know the communities’ level of adherence on COVID-19 prevention measures. Thus, this study aimed to identify the predictors of adherence to COVID-19 prevention measure among communities in North Shoa zone, Ethiopia by using a health belief model.

**Methods:**

Community-based cross-sectional study design was employed. A total of 683 respondents were interviewed using a structured and pre-tested questionnaire. The data were collected by using a mobile-based application called “Google form.” Logistic regression was performed to analyze the data. Estimates were reported in adjusted odds ratios with 95% confidence intervals (CI) and a significant association was declared at p-value of less than 0.05.

**Result:**

The overall adherence level of the community towards the recommended safety measures of COVID-19 was 44.1%. Self-efficacy (AOR = 0.23; 95% 0.14, 0.36), perceived benefits (AOR = 0.35; 95% 0.23, 0.56), perceived barriers (AOR = 3.36; 95% 2.23, 5.10), and perceived susceptibility of COVID-19 (AOR = 1.60; 95% 1.06, 2.39) were important predictors that influenced the adherence of the community to COVID-19 preventive behaviors.

**Conclusions:**

In this study, the overall adherence level of the community towards the recommended safety measures of COVID-19 was relatively low. It is vital to consider the communities’ self-efficacy, perceived benefits, perceived barriers and perceived susceptibility of COVID-19 in order to improve the adherence of the community towards the recommended safety measures of COVID-19.

## Introduction

Coronavirus disease 2019 (COVID-19) is an emerging respiratory infections and is known to cause illness ranging from the common cold to severe acute respiratory syndrome. COVID-19 is known to spread from human-to-human through droplets and direct contact. In December 2019, a novel corona virus (COVID-19) was detected in three patients with pneumonia connected to the cluster of acute respiratory illness cases from Wuhan, China [1]. The pathogen of this disease was confirmed as a novel corona virus by molecular methods and was initially named as 2019 novel corona virus (2019-nCoV); however, on January 30, 2020, the WHO declared the COVID-19 outbreak as the sixth public health emergency of international concern. Therefore, this outbreak constitutes a public health risk through the international spread of disease and requires a coordinated international response. Increased dissemination of information through use of the internet is associated with increased transmission of information from all geographical regions and across disciplines regarding recognition of COVID-19 [2].

Corona viruses are single-stranded RNA, non-segmented, enveloped viruses, that cause illness ranging in severity from the common cold to severe and fatal illness [3]. Robust estimates for final case fatality risk for COVID-19 are still lacking and biased due to incomplete outcome data [4].

To date, there are no vaccines that protect COVID-19. The best way to prevent infection is avoiding exposure to this virus [5]. Knowledge and implementation of the community on the measures recommended by Communicable Disease Control (CDC) and World Health Organization (WHO) for both prevention and case finding are crucial [6].

The most commonly reported clinical symptom in laboratory-confirmed cases were fever (88%), followed by dry cough (68%), fatigue (38%), sputum production (33%), dyspnea (19%), sore throat (14%), headache (14%) and myalgia or arthralgia (15%) [7]. Less common symptoms were diarrhea (4%) and vomiting (5%) [8]. About 80% of reported cases in China had mild to moderate disease, 13.8% had severe disease and 6.1% were critical [9]. Current estimates suggest a median incubation period from five to six days for COVID-19, with a range from one to up to 14 days [10].

As of December 2019 until August 27, 2020, 23:42 GMT, a total of 24,603,578 confirmed cases, including 61,536 with severe illness, 17,072,656 recovery and 834,719 deaths had been reported worldwide. In Ethiopia, there were 46,407 confirmed cases, including 330 with severe illness, 16,829 recovery and 745 deaths reported since March 13 to August 27, 2020, 23:42 GMT. To date (August 27/2020), COVID-19 has affected people in more than 215 countries, including Ethiopia, and has become a global threat. America has the largest number of patients with COVID-19 (6,044,411), followed by Brazil (3,764,493) and India (3,384,575). Ethiopia was 52 ranking country on the world level. However, asymptomatic patients or patients with mild COVID-19 symptoms may not seek health care, nor receive diagnosis, which leads to underestimation of the burden of COVID-19. African countries with the highest estimated numbers of COVID-19 cases were South Africa, Egypt, Morocco, Nigeria and Ethiopia [11]. 54.3% of those infected with SARS-CoV-2 were male with a median age of 56 years. Patients who required intensive care support were older and had multiple comorbidity such as cardiovascular, cerebrovascular, endocrine, digestive, and respiratory disease. Those in intensive care were also more likely to report dyspnea, dizziness, abdominal pain, and anorexia [12].

The impact of the COVID-19 outbreak on the East and Horn of Africa is expected to be far- reaching and more catastrophic than for high income countries, These pre-existing conditions such as the population size, status of health systems and health workforce are expected to worsen any health impacts from COVID-19 [13, 14].

Ethiopia is the second most populous nation in Africa, and home to close to 9% of the African population. Ethiopia respond to COVID-19 started early. On January, the government introduced passenger-screening protocols at Addis Ababa’s international airport, and further preparation continued in January and February. The first report of a COVID-19 case in Ethiopia was on March 13, two days after the global pandemic was declared. National responses were scaled up soon after, and a state of emergency was declared on April 8 [15]. In response to COVID-19, the federal and regional governments in Ethiopia have been taking a series of policy actions. These include closing schools, restricting use of public transportation, banning large meetings, and suspending sporting and religious gatherings [16].

The following basic measures to reduce transmission of COVID-19 have been recommended by the WHO and have been adopted by the Ethiopian Government, such as wash hands frequently using soap, maintain social distancing, stay informed and follow advice given by healthcare provider, stay at home. If you begin to feel sick and if you develop fever or cough or experience difficulty breathing, seek medical advice and call in advance the center. Even though COVID-19 case diagnosis and knowledge on prevention remain vital components of the control strategies, little is known about the knowledge, risk perception and perceived self-efficacy of the community in the context of Ethiopia. The Ethiopian government is expanding its diagnosis system in order to increase accessibility of COVID-19 diagnosis and treatment to regional and zonal levels. There is no study to date that has been conducted to assess the knowledge of the community on prevention, risk perception and evaluate the implementation of policies and regulations set for the prevention and control of COVID-19 by the community in Ethiopia. Evidence on this subject would contribute to the prevention, control of COVID-19 as well as on the management of its impact. Therefore, this cross-sectional survey is aimed to assess the predictors of adherence COVID-19 prevention measure among communities in North Shoa Zone, Ethiopia based on health belief model. The Health Belief Model (HBM) provided the theoretical base for this study. The HBM was developed by psychologists in the United State Public Health Service in the 1950s as a 6 way to explain participation in medical prevention and disease detection programs [17]. The model suggests that changes in preventive health behaviour are originally based on six factors, susceptibility: perceived personal vulnerability to or subjective risk of a health condition, seriousness: perceived personal harm of the condition, benefits: perceived positive attributes of an action, barriers: perceived negative aspects related to an action, health motivation refers to beliefs and behaviours related to the state of general concern about health and confidence is defined as the belief that one can successfully perform a behaviour that will then lead to a desirable outcome [18, 19]. The aim of this model is to increase the perception of individuals about a health threat and direct their behaviors towards health. Likewise, it focuses on a person’s health related behavior and belief in predicting future actions. The perceived risk of people developing COVID 19 is considered to be the primary motive to change within the Health Belief Model, which assumes that the higher the perceived threat, the more likely an individual will modify his or her behavior to avoid that threat.

## Methods

### Study setting and period

The study was conducted from May 1–30 /2020 at North Shoa zone, Amhara regional state, Ethiopia. Debre Berhan town (the zone city) is located 130 kilometers far from Addis Ababa (the capital city of Ethiopia). The zone has 21 rural districts and 3 city administrations; 370 rural kebeles, 523,338 households; 370 health posts; and 740 health extension workers. The zone has 1 comprehensive, specialized referral hospital, 7 primary hospitals, 95 health centers, 389 health posts, 2 private hospitals, 34 private medium level clinics, 97 private primary clinics, 8 pharmacies and 1 private rural drug vendor (*unpublished zonal health department report*, *2020)*.

### Study design

A community-based cross-sectional study design was employed to identify predictors of adherence to COVID 19 prevention measures among communities using the health belief model.

### Sample size determination and sampling procedures

The sample size was determined by using single population proportion formula. The following assumption was made: margin of error was 5%, confidence interval was 95%, the proportion of adherence was 50%, design effect was 1.5 and non-response rate was 10%. In this study, a multi-stage sampling technique was applied. Initially, the zone was stratified into two strata: urban and rural districts. Thirty percent of the districts were selected from each stratum (seven from rural district and two from urban districts). In the second stage, a total of 27 kebeles were selected randomly. At kebele level, family folders (list of households taken from health extension workers) were used as the sampling frame and the required numbers of households were selected using systematic random sampling technique. The first household was selected by using a lottery method, and then every 6^th^ household was included in the study. If data collectors could not find both of the household heads with three visits, they shifted to the next immediate household.

### Instrument and measurement

Data was collected by “Google forms”. A structured questionnaire was adopted from a WHO survey tool for COVID-19 [20]. The questionnaire was prepared first in English and translated to Amharic and back translated to English before data collection process. The questionnaire had four parts: socio-demographics variables, health belief model constructs, adherence of the community towards COVID-19 prevention measures and knowledge of the community towards COVID-19. Health belief model constructs were a 5-likert scale item (1 = strongly dissatisfied, 5 = strongly dissatisfied), then during analysis strongly dissatisfied and dissatisfied merged to disagree and strongly agree and agree merged to agree. Similarly, the adherence level of the community towards COVID-19 prevention measures were a 5-likert scale item (1 = never, 2 = rarely, 3 = sometimes, 4 = often, 5 = always). Higher scores indicate greater safety measure practice, while lower scores indicate less safety measure practice. Survey questions were tested for content validity and internal validity (reliability). The content validity of questionnaire was evaluated by public health and nursing expertise. Based on their endorsements, modifications to the survey tool were made. Moreover, the Cronbach alpha coefficients of HBM constructs were calculated. The reliability scores are presented in Table 1.

**Table 1:** Reliability score of attitudinal aspects of questions towards HIV/AIDS and STIs scale.

Scale	Cronbach’s alpha value
Perceived susceptibility	0.57
Perceived severity	0.82
Perceived benefits	0.91
Perceived barriers	0.84
Cues to action	0.68

In addition, the reliability score (cronbach alpha) of adherence tool was 0.91. Based on these questions, mean scores of perceived susceptibility, perceived severity, perceived benefits, perceived barriers and cues to action were calculated to classify the respondents into two groups; above the mean and below the mean.

### Data collection

Data was collected by 27 health care workers. Google platform was used to collect data. The Universal Resource Locator (URL) address was sent to each data collector through their mobile telegrams. Training was provided for data collectors and supervisors on the objective of the study, data collection procedures, COVID-19 precaution, and ethical considerations.

### Data analysis

Data were checked for completeness and inconsistencies. The online datasheet was downloaded from the Google form in excel format and then exported to SPSS version 21 software for further analysis. Descriptive statistics (mean, median, standard deviation, Inter Quartile Range, percentage, and frequency distribution) were computed. Logistic regression was performed to analyze the data. Those independent variables which were statistically significant in the bivariate model (*p-value < 0*.*05*) were entered into the multivariable analysis. In the final model, a significant association was declared at a p-value of less than 0.05. The results were presented in texts and tables with adjusted odds ratio (AOR) and the corresponding 95% CI.

### Ethical approval and considerations

Ethical clearance was obtained from the Institutional Review Board (IRB) of the Debre Berhan University. Written informed consent was obtained from each participant. All the information obtained from the study participants were kept confidential throughout the process of study, and the name of the participant was replaced by code. Withdrawal from the study at any point if they wished was assured.

## Results

### Socio-demographic characteristics of participants

Six hundred eighty three respondents were participated in this study. The majority, 390 (57.1%) of participants were male. The median age of the respondents was 38 (IQR 30–48 years). A high number of the respondents 492 (72%) were married. Three hundred forty eight (51%) of the respondents did not attend formal education. More than half of the respondents, 377 (55.2%) were from rural and 251 (36.7%) were farmers. Most of them, 566 (82.9%) had no chronic illness (Table 2).

**Table 2:** Socio demographic characteristics of participants, North Shoa zone, Ethiopia, 2020.

Variables	Category	Frequency	Percentage
Age	<39	364	53.3%
	40–59	265	38.8%
	>60	54	7.9%
Sex	Male	390	57.1%
	Female	293	42.9%
Marital status	Single	113	16.5%
	Married	492	72.0%
	Separated	11	1.6%
	Divorced	32	4.7%
	Widowed	35	5.1%
Religion	Orthodox	677	99.1%
	Protestant	6	0.9%
Ethnicity	Amhara	679	99.4%
	Other	4	0.6%
Educational Status	No Formal Education	348	51.0
	Primary	123	18
	Secondary	101	14.8
	Higher education	111	16.3
Occupational status	Governmental org	94	13.8%
	Private organization	95	13.9%
	Merchant	73	10.7%
	Farmer	251	36.7%
	House wife’s	117	17.1%
	Daily worker	19	2.8%
	Student	21	3.1%
	Other	13	1.9%
Place of residence	Urban	306	44.8%
	Rural	377	55.2%
Monthly income	Low	398	58.3%
	Medium	267	39.1%
	High	18	2.6%
History of chronic Illness	Yes	102	14.9%
	No	566	82.9%
	I do not know	15	2.2%
Medical professional	Yes	21	3.1%
	No	662	96.9%

### Knowledge of the community towards COVID-19

The mean knowledge of the participants towards COVID-19 was 10 with (SD ±2.66). Nearly, half of participants (47.1%) had inadequate knowledge of COVID-19.

### Health belief model constructs for COVID-19

#### Perceived sensibility

The overall perceived susceptibility of COVID-19 infection was calculated using the mean score. The mean score of perceived susceptibility of COVID-19 was 13.88 (±3.38 SD). Forty-five percent (44.8%) of the respondents were above the mean score, and they perceived themselves susceptible to COVID-19 (Table 3).

**Table 3:** The respondents’ perceived susceptibility to COVID-19 infection in North-shoa zone, Ethiopia.

Variables	Disagree	Neutral	Agree
I am susceptible to COVID-19	162(23.7)	31(4.5)	490(71.0)
Only old people are susceptible to COVID-19	504(73.8)	19(2.8)	160(23.4)
Only person living urban is susceptible to COVID-19	565(82.7)	18(2.6)	100(14.6)
Any sex groups are susceptible to COVID-19	137(20.1)	11(1.6)	535(78.3)
Religious persons are not being affected by COVID-19 infections	343(50.2)	37(5.4)	303(44.4)
I don't care about this disease and do my daily activities like before	352(51.5)	27(4.0)	304(44.5)
Catching or not catching the COVID-19 infection is out of your control	267(39.1)	58(8.5)	358(52.4)
**Perceived susceptibility (mean = 13.88)**	**Not susceptible**	**Susceptible**	
	**377 (55.2)**	**306 (44.8)**	

#### Perceived severity of COVID-19

Perceived severity of COVID-19 infection was measured with five questions. The highest number of participants (83.7) concerned about the severity of COVID-19 infection.

#### Perceived barriers of COVID-19 preventive measure

In our study area, the study participants pointed out their perceived barriers of COVID-19 infection prevention. As demonstrated in Table 4, more than half of participants (55.6) do not have barriers of COVID-19 infection prevention.

**Table 4:** The respondents’ perceived barriers of COVID-19 infection prevention in North-shoa zone, Ethiopia, 2020.

Variables	Disagree	Neutral	Agree
I have no knowledge how to prevent COVID-19	545(79.8)	44(6.4)	94(13.8)
It is difficult to find water and soap at any place	329(48.2)	27(4.0)	327(47.8)
I do not wash my hands because, it takes long time	604(88.4)	19(2.8)	60(8.8)
I do not wear face masks because the mask is scarce in the market.	293(42.9	60(8.8)	330(48.3)
I do not use alcohol based sanitizers because it’s scarcity in the market.	294(43.0	54(7.9)	335(49.0)
It is difficult not to touch hands, mouth, nose and eyes	281(41.1)	26(3.8)	376(55.1)
Staying at home to prevent the disease is difficult	249(36.5)	21(3.1)	413(60.5)
Washing hands repeatedly costs much and me and my families will not afford	575(84.2)	18(2.6)	90(13.2)
It is hard to remember washing hands repeatedly.	476(68.4)	16(2.3)	200(29.3)
I cannot stop shaking hands because my relationships with people become affected	536(78.5)	15(2.2)	132(19.3)
I cannot stop going to religious places because my God protect me from any diseases.	320(46.9)	35(5.1)	328(48)
I cannot keep physical distancing because it is difficult	472(69.1)	29(4.2)	182(26.6)
**Perceived barriers for preventive measure (mean = 20.93)**	**Have not barriers**	**Have barriers**	
	**368 (53.9)**	**315 (46.1)**	

#### Perceived benefit of preventive safety measures of COVID-19

Our study participants also reported the benefit of preventive safety measures of COVID-19 infection. Consequently, nearly three-fourth of our respondents (72.0%) agreed with the usefulness of practicing the recommended safety measures to prevent COVID-19 infection (Table 5).

**Table 5:** The respondents’ perceived benefits of safety measures to prevent COVID-19 infection in North-shoa zone, Ethiopia, 2020.

Variables	Disagree	Neutral	Agree
I believe that hand washing is helpful for me to prevent myself from COVID-19	20(2.9)	8 (1.2)	655(95.9)
I believe that social distancing is helpful for me to prevent myself from COVID-19	24(3.3)	15 (2.2)	644 (94.3)
I believe that staying at home is helpful for me to prevent myself from COVID-19	42(6.1)	18 (2.6)	623 (91.2)
I believe that avoiding from overcrowding place is helpful for me to prevent myself from COVID-19	18(2.6)	20(2.9)	645(94.4)
I believe that praying out of religion place/home is helpful for me to prevent myself from COVID-19	73(10.7)	22 (3.2)	588(86.1)
I believe that the government restriction is helpful for me to prevent myself from COVID-19	44 (6.4)	15(2.2)	624(91.4)
I believe that the information regarding COVID-19 is helpful for me to prevent myself from COVID-19	23 (3.4)	14(2.0)	646(94.6)
I believe that stop shaking people’s hand is helpful for me to prevent myself from COVID-19	27 (4.0)	15(2.2)	641(93.9)
I believe that respecting the rules and regulations set by government is helpful for me to prevent myself from COVID-19	17(2.5)	11(1.6)	655(95.9)
I believe that washing my hands after coughing or sneezing, or doing something is helpful to cure myself and my families.	17(2.5)	25(3.7)	641(93.9)
**Perceived benefits** of safety measures (mean-28.86)	Not useful	Useful	
	**293 (42.9)**	**390 (57.1)**	

#### Cues to action to COVID-19

There were signals in the community to act upon the prevention of COVID-19 infections. Accordingly, more than eighty percent of our respondents (84.3%) had cues to act on COVID-19 infection prevention.

### The adherence level of the community towards COVID-19 safety measures

The overall adherence level of the community towards the recommended safety measures of COVID-19 was 44.1% (95% CI = 41.1, 48.2). Only nine percent of participants did not practice handwashing with soap and 42.2% of the respondents did not utilize sanitizers to clean hand (Table 6).

**Table 6:** Level of adhering to recommended safety measures of COVID-19 among the communities of North Shoa zone, Ethiopia 2020.

Safety measures	Level of taking recommended COVID-19 safety measures				
	Never (%)	Rarely (%)	Sometimes (%)	Often (%)	Always (%)
I have practiced recommend hand washing for at least 20 seconds	63 (9.3)	136 (19.9)	196 (28.7)	163 (23.9)	125 (18.3)
I have practiced avoiding touching eyes, nose, and mouth with unwashed hands	68 (10.0)	133 (19.5)	216 (31.6)	176 (25.8)	90 (13.2)
I have practical use of disinfectants to clean hands when soap and water was not available.	288 (42.2)	112 (16.4)	94 (13.8)	91 (13.3)	98 (14.3)
I have been staying at home when I was sick or when I had a cold	132 (19.3)	183 (26.8)	167 (24.5)	116 (17.0)	85 (12.4)
I have been practicing covering my mouth and nose when I cough or sneeze	49 (7.2)	111 (16.3)	127 (18.6)	227 (33.2)	169 (24.7)
I have been practicing physical distancing at least 2-meter away from others.	126 (18.4)	157 (23.0)	114 (16.7)	159 (23.3)	127 (18.6)
I have been practicing self-isolation when I have a fever, cough and headache	137 (20.1)	163 (23.9)	135 (19.8)	136 (19.9)	112 (16.4)
I have been practicing disinfecting surfaces that belongs to me.	259 (37.9)	104 (15.2)	100 (14.6)	100 (14.6)	120 (17.6)
I have been wearing a face mask when I go to crowding area	374 (54.8)	90 (13.2)	57 (8.3)	69 (10.1)	93 (13.6)
I am disinfecting my mobile phone with alcohol based sanitizer	371 (54.3)	102 (14.9)	74 (10.8)	62 (9.1)	74 (10.8)
**Preventive behaviors**	**Poor preventive behavior**			**Good preventive behavior**	
	**377 (55.2)**			**306 (44.8)**	

### Factors associated with the adherence of the community towards COVID-19 safety measures

The odds of adherence to safety measures of COVID-19 were 1.60 times higher among communities who perceived that they were not susceptible to COVID-19 than their counterparts (AOR: 1.60, 95%CI: 1.06, 2.39). Members of the community who did not have a perception of barriers of COVID-19 measures was 3.36 times higher on poor adherence of COVID-19 recommended preventive measures as compared to those who had a perception of barriers of COVID-19 measures (Table 7).

**Table 7:** Multivariate analysis of adherence status on recommended safety measures of COVID-19 among community in North Shoa zone, Ethiopia 2020.

Variable	Adherence status		COR (95%CI)	AOR (95%CI)
	Poor adherence	Good adherence		
**Age of respondent**				
18–39	174(25.4)	190 (27.8)	1.86(1.03, 3.35)	1.53 (0.72, 3.25)
40–59	169 (24.7)	96 (14.1)	0.97(0.53, 1.77)	0.97 (0.45, 2.06)
> = 60	34 (5.0)	20 (2.9)	1.00	1.00
**Educational status**				
No formal education	219 (32.1)	129 (18.9)	1.00	1.00
Primary school	65 (9.5)	58 (8.5)	1.52(1.01, 2.30)	0.96 (0.56, 1.66)
Secondary school	52 (7.6)	49 (7.2)	1.60(1.02, 2.50)	0.77 (0.42, 1.43)
Certificate and above	41 (6.0)	70 (10.2)	2.90(1.86, 4.51)	1.08 (0.46, 2.53)
**Occupation**				
Government	34 (5.0)	60 (8.8)	1.00	1.00
Private organization	86 (12.6)	82 (12.0)	0.54(0.32, 0.91)	0.51 (0.22, 1.19)
Farmer	224 (32.8)	144 (21.1)	0.36(0.23, 0.58)	0.53 (0.22, 1.30)
Day laborer	14 (2.0)	5 (0.7)	0.20(0.06, 0.61)	0.22 (0.05, 1.01)
Students	19 (2.8)	15 (2.2)	0.45(0.20, 0.99)	0.49 (0.15, 1.57)
Monthly income				
< = 2,040	236(34.6)	162 (23.7)	0.26(0.09, 0.76)	0.28 (0.07, 1.21)
2,040–10,200	136 (19.9)	131 (19.2)	0.37(0.13, 1.07)	0.32 (0.08, 1.36)
> 10,200	5 (0.7)	13 (1.9)	1.00	1.00
Perception on susceptible of COVID-19				
Not susceptible	165 (24.2)	212 (31.0)	2.90(2.11, 3.98)	1.60 (1.06, 2.39)*
Susceptible	212 (31.0)	94 (13.8)	1.00	1.00
Perception on severity of COVID-19				
Not sever	196 (28.7)	123 (18.0)	0.68(0.46, 0.84)	1.11 (0.73, 1.69)
Sever	181 (26.5)	183 (26.8)	1.00	1.00
Perception of usefulness of safety measures				
Not useful	209(30.6)	84 (12.3)	0.30(0.22, 0.42)	0.35 (0.23, 0.53)*
Useful	168 (24.6)	222 (32.5)	1.00	1.00
Perception of barriers of COVID-19 measures				
Have no barrier	129 (18.9)	239 (35.0)	6.86(4.86, 9.68)	3.36 (2.23, 5.10)*
Have barrier	248 (36.3)	7 (9.8)	1.00	1.00
Cues to action on COVID-19				
Have no cues to action	78 (11.4)	29 (4.2)	0.40(0.25, 0.63)	1.16 (0.65, 2.06)
Have cues to action	299 (43.8)	277 (40.6)	1.00	1.00
Self-efficacy				
Have no self-efficacy	202 (29.6)	39 (5.7)	0.12(0.09, 0.02)	0.23 (0.14, 0.36)*
Have self-efficacy	175 (25.6)	267 (39.1)	1.00	1.00

* Significant at P < 0.05

## Discussions

COVID-19 pandemic has created fear circumstances of the human race globally. The impact progress to social activities, education, health services and other services were affected [21]. Ethiopia has implemented preventive guidelines recommended by WHO to protect human to human transmission of COVID-19, such as social distancing, ban on public gathering, religious gatherings, regular personal hygiene, use of face masks, cover the mouth and nose while sneezing or coughing, one per seat in public vehicles, temporary closing of schools, Colleges and Universities [22].

The achievement of the world’s fight against COVID-19 depends upon people’s adherence to the control measures [23, 24]. The battle against COVID-19 in Ethiopia is still in its infancy. The first confirmed case was announced on March 13/2020. The community’s adherence to these control measures is largely affected by their health belief towards COVID19 and its preventive measure. Since Ethiopian society is vulnerable to COVID-19, this health belief on risk perception gap is potentially dangerous and should be addressed to mitigate the disease spread.

The HBM implies that an individual’s perception of his/her susceptibility to a disease, attached to his/her belief that the disease has potentially serious consequences (perceived seriousness) equals the perceived threat which leads to a behavior such as using safety measure and testing. If the person believes that behaviors such as using safety measure are beneficial and outweigh his/her perceived barriers, then he/she is more likely to adopt the new behavior. Cues to action are events, people or things that encourage people to change their behavior. Examples of agents of cues to action include family, friends, media, and health care providers. Other modifying variables include age, gender, ethnicity, personality, socioeconomic, knowledge and motivation [17].

Findings from this study showed that 102(14.9%) participants were had a chronic disease, whereas study done at Turkish showed that 54 (15.7%) participants had a chronic disease [25].

According to the current study, the mean knowledge score was 10 with (SD ±2.66). More than half 361(52.9%) of the respondents had adequate knowledge on COVID-19. A study done in Pakistan medical students showed that, 80% of participants had sufficient knowledge about coronavirus [26]. A study done in Bangladesh showed that, the mean knowledge score for participants was 10.77 (SD ±0.588). Around 82.85% of the participants were having adequate knowledge on COVID 19 and other finding, 61.2% participants had adequate knowledge to COVID 19 [27, 28]. A National Survey of Syrian indicated that, 75.6% Participants were shown a good level of awareness regarding COVID-19 [29]. Finding from Chinese resident, the mean COVID-19 knowledge score was 10.8 (SD±1.6), around 90% of participants were knowledgeable [30]. Study done on quarantine population in Tigrie region, Ethiopia showed that, the mean knowledge score was 8.73 (±2.64). Less than half, 42.9% of the study participants were knowledgeable [31].

Regarding perceived susceptibility, forty-five percent (44.8%) of the respondents were above the mean score, and they perceived themselves susceptible to COVID-19 infection. While a cross-sectional study in Myanmar showed that, 77.2% were had moderate risk perception and 22.8% were had high risk perception [32]. Study done South Korea showed, a high proportion of the respondents were perceived the seriousness of COVID-19 [33]. Cross-sectional studies in America, 50% of study participants were perceived the risk of COVID 19 [34]. A worldwide cross-sectional study across the ten sampled countries, risk perception were varied between 4.78 and 5.45 [35].

Regarding to the perceived severity of COVID-19 infection, 572 (83.7) participants were perceived severity of COVID-19 infection. Study done in India 90% of the participants considered COVID-19 as a serious disease [36]. Study in Egypt and China, most participants were believed that COVID-19 is a life-threatening, so any member of the families have risk of infection [37], whereas 80% of china’s were believed that COVID 19 severely harms health [38].

In this study, more than half of participants (55.6) did not have barriers to implement to COVID-19 prevention measures, whereas study in Egypt, more than two fifths (22.7%) of study participants were believed that infection is associated with stigma [37]. In our Study, nearly three-fourth of the study respondents (72.0%) was agreed on the usefulness of practicing the recommended safety measures. The global based study conducted in ten selected countries showed that, the act of practicing recommended safety measure for the greater benefit of society is relevant [35].

The respondents’ belief of COVID-19 infection was formulated from HBM constructs. As a result, eighty-four percent of the respondents have cues to perform prevention measure activities towards COVID-19 and sixty-four percent of our study participants have self-efficacy to apply preventive measures of COVID-19 infections. This result indicated that there are still gaps on the belief of COVID-19 infection. Since Ethiopian communities have a strong societal ties and poor socioeconomic basis which are now risk factors for COVID-19 transmission [39, 40], the communities need to ensure self-efficacy to apply preventive measures of COVID-19 infections.

This study suggests that, 93 (13.6%) participants were always wearing a mask, 125 (18.3%) were always washing hands with soap for 20 seconds, 90 (13.2%) participants were always practiced avoiding touching eyes, nose, and mouth with unwashed hands, 85 (12.4%) were always staying at home, when have cold or fever and 127 (18.6%) were always applying recommended social distancing. The overall level of adherence to recommended safety measures was 44.8%. The study done in India, showed that 93.2% of the participants have adopted preventive measures to control COVID-19 and 97.6% were wear a mask, 97.3% were frequently wash hands, 97.8% were avoid handshaking and hugging, and 95% were avoid going out [36]. Another study done in Bangladesh, showed that 87.97% of participants were followed social distancing in their daily life [41]. Community based study Done at Pakistan showed that, 92.8% of respondents were practiced maintaining safe physical distance [42]. In Punjab 71.8% participants were always wearing a mask when going outside, 91.8% participants were followed the stay at home policy, 53.8% participants were followed the steps of hand hygiene [43]. Based on an impact survey done America, about 94.7% of participants reported washing or sanitizing hands, 39.6% reported working from home, and 9.6% reported visiting a doctor or hospital [44]. Survey of Bangladish showed that, more than half 51.6% of respondents had good practices of COVID 19 preventive measure [28]. Study done on quarantine population in Tigrie region, Ethiopia showed that, nearly half, 165 (49.8%) of the participants have gone to crowded places, Forty-six percent of the participants did not use a face mask when leaving home, more than half, 54.4% of participants did not obey the preventive measures given by local health care authorities [31]. A study done in US, more than half of patients (58.6%) were reported that the corona virus caused them to change their daily routine activities [45] other study done in China, majority of the 96.4% participants were had not visited any crowded place and 98.0% participants were wore masks when going out [30, 45]. Moreover, the reported prevalence of adherence level of community was likely to be conservative as this was based on community recall and self-reports which usually overestimate or underestimate community adherence levels. The cultural and socioeconomic characteristics of the community also determine the community adherence level. Here in the study area the communities are highly socially bonded by “Edir”, “Ekub” and funereal ceremonies any other social structures which may affect the adherence level of the community on recommended safety measures.

Regarding with factors, community perception on the susceptibility of COVID-19 measures, barriers on prevention measures of COVID-19 and the presence of self-efficacy showed significant association with level of recommended safety measures adherence on COVID-19.

Poor adherence of recommended safety measures of COVID-19 were 38% less likely among household members who share a common bed compared from the counterpart. This was available evidence implies that, at least 1 meter physical distancing was associated with a large reduction in infection, and distances of 2 meters were more effective. These data also suggest that wearing face masks protect people (both healthcare workers and the general public) against infection by corona viruses, and that eye protection could confer additional benefit (3). These may be possibly due to that if family members share a common bed they may face difficulty in keeping social distancing.

The odds of poor adherence on recommended safety measures of COVID-19 were 1.60 times higher among communities who perceives they are not susceptible for COVID-19 than the counterpart (AOR: 1.60, 95%CI: 1.06, 2.39). A Community that has no barriers on preventive measures were 3.36 times higher on poor adherence of COVID-19 recommended preventive measures. Community members who had self-efficacy 77% more likely adherence to COVID 19 safety measure.

## Conclusions

In this study, the overall adherence level of the community towards the recommended safety measures of COVID-19 was relatively low. It is vital to consider the communities’ self-efficacy, perceived benefits, perceived barriers and perceived susceptibility of COVID-19 in order to improve the adherence of the community towards the recommended safety measures of COVID-19.

## Limitation of the study

This study has different limitations. Firstly, this study followed a cross-sectional study design that cannot establish causal inferences. Secondly, were used face-to-face interviews to collect data, which is prone to social desirability biases. Thirdly, there may be sample selection biases. There was no previously standardized and validated tool to assess the Knowledge and community health belief towards COVID-19. The area is not well studied, so we didn’t find adequate studies to compare and contrast our findings with others, and it makes our discussion narrow. Despite these limitations, the study finding provides valuable information about the adherence level of the communities towards the recommended safety measure of COVID 19.

## Supporting information

S1 Annex English version of the survey questionnaire. (DOCX)

## Abbreviations

AOR: Adjusted Odds Ratio
CDC: Communicable Disease Control
CI: Confidence Interval
COVID-19: Coronavirus Infectious Disease-19
COR: Crude Odds Ratio
HBM: Health Belief Model
IRB: Institutional Review Board
SARS-CoV-2: Severe Acute Respiratory Syndrome Coronavirus 2
SPSS: Statistical Package for the Social Sciences
URL: Universal Resource Locator
WHO: World Health Organization
2019-nCoV: 2019 Novel Coronavirus

## References

[1] Assessment RR: Coronavirus disease 2019 (COVID-19) in the EU/EEA and the UK–ninth update. In.: European Centre for Disease Prevention and Control: Stockholm 2020.

[2] Lai C-C, Shih T-P, Ko W-C, Tang H-J, Hsueh P-R: Severe acute respiratory syndrome coronavirus 2 (SARS-CoV-2) and corona virus disease-2019 (COVID-19): the epidemic and the challenges. *International journal of antimicrobial agents* 2020:105924 10.1016/j.ijantimicag.2020.105924 32081636 PMC7127800

[3] Costantine MM, Landon MB, Saade GR: Protection by Exclusion: Another Missed Opportunity to Include Pregnant Women in Research During the Coronavirus Disease 2019 (COVID-19) Pandemic. *Obstetrics and Gynecology* 2020 10.1097/AOG.0000000000003924 32349053 PMC7219859

[4] Siordia JA Jr: Epidemiology and clinical features of COVID-19: A review of current literature. *Journal of Clinical Virology* 2020:104357 10.1016/j.jcv.2020.104357 32305884 PMC7195311

[5] Covid C, Team R: Severe outcomes among patients with coronavirus disease 2019 (COVID-19)—United States, February 12–March 16, 2020. *MMWR Morb Mortal Wkly Rep* 2020, 69(12):343–346. 10.15585/mmwr.mm6912e2 32214079 PMC7725513

[6] Organization WH: Novel Coronavirus (‎ 2019-nCoV)‎: situation report, 3. 2020.

[7] Wang J, Zhou M, Liu F: Reasons for healthcare workers becoming infected with novel coronavirus disease 2019 (COVID-19) in China. *J Hosp Infect* 2020, 20 10.1016/j.jhin.2020.03.002 32147406 PMC7134479

[8] Peyronnet et al: Infection par le SARS-CoV-2 chez les femmes enceintes. État des connaissances et proposition de prise en charge. CNGOF. *Gynécologie Obstétrique Fertilité & Sénologie* 2020. 10.1016/j.gofs.2020.10.001 PMC7534662 33031963

[9] Chen et al: Clinical characteristics and intrauterine vertical transmission potential of COVID-19 infection in nine pregnant women: a retrospective review of medical records. *The Lancet* 2020, 395(10226):809–815. 10.1016/S0140-6736(20)30360-3 32151335 PMC7159281

[10] Xu et al: Epidemiological data from the COVID-19 outbreak, real-time case information. *Scientific data* 2020, 7(1):1–6. 10.1038/s41597-019-0340-y 32210236 PMC7093412

[11] Organization WH: Risk communication and community engagement (‎ RCCE)‎ readiness and response to the 2019 novel coronaviruses (‎ 2019-‎‎ nCoV)‎‎‎: interim guidance, 26 January 2020. 2020.

[12] Bai et al: Presumed asymptomatic carrier transmission of COVID-19. *Jama* 2020, 323(14):1406–1407. 10.1001/jama.2020.2565 32083643 PMC7042844

[13] Peterman et al: Pandemics and violence against women and children. *Center for Global Development working paper* 2020, 528.

[14] Council ADPA: Pediatric preparedness resource kit. 2013.

[15] Abate GT, de Brauw A, Hirvonen K: Food and nutrition security in Addis Ababa, Ethiopia during COVID-19 pandemic: June 2020 report, vol. 145: Intl Food Policy Res Inst; 2020.

[16] Baye K: COVID-19 prevention measures in Ethiopia: Current realities and prospects, vol. 141: Intl Food Policy Res Inst; 2020.

[17] Glanz K, Rimer BK, Viswanath K: Health behavior and health education: theory, research, and practice: John Wiley & Sons; 2008.

[18] Ahmed BaA: Awareness and practice of breast cancer and breast-self examination among university students in Yemen. *Asian Pacific journal of cancer prevention*: *APJCP* 2010, 11(1):101–105. 20593937

[19] Griffin JL, Pearlman MD: Breast cancer screening in women at average risk and high risk. *Obstetrics & Gynecology* 2010, 116(6):1410–1421. 10.1097/AOG.0b013e3181fe714e 21099612

[20] Organization WH: Survey tool and guidance: rapid, simple, flexible behavioural insights on COVID-19: 29 July 2020. In.; 2020.

[21] Kassema J: COVID-19 outbreak: is it a health crisis or economic crisis or both. *Case of African Counties Case of African Counties (23 Mar*, *2020)* 2020.

[22] Harris et al: The effect of COVID-19 and government response measures on poor and vulnerable groups in urban areas in Ethiopia. In.: Research Report: Results from the first round of a mixed method panel study …; 2020.

[23] Ajilore K, Atakiti I, Onyenankeya K: College students’ knowledge, attitudes and adherence to public service announcements on Ebola in Nigeria: Suggestions for improving future Ebola prevention education programmes. *Health Education Journal* 2017, 76(6):648–660.

[24] Tachfouti N, Slama K, Berraho M, Nejjari C: The impact of knowledge and attitudes on adherence to tuberculosis treatment: a case-control study in a Moroccan region. *Pan African Medical Journal* 2012, 12(1). PMC3428172 22937192

[25] Özdin S, Bayrak Özdin Ş: Levels and predictors of anxiety, depression and health anxiety during COVID-19 pandemic in Turkish society: The importance of gender. *International Journal of Social Psychiatry* 2020:0020764020927051. 10.1177/0020764020927051 32380879 PMC7405629

[26] Ikhlaq A, Hunniya B-E, Riaz IB, Ijaz F: Awareness and attitude of undergraduate medical students towards 2019-novel Corona virus. *Pakistan Journal of Medical Sciences* 2020, 36(COVID19-S4):S32 10.12669/pjms.36.COVID19-S4.2636 32582311 PMC7306955

[27] Hossain I, Ahmad SA, Khan MH, Rahman A: COVID-19 AND CHANGING BEHAVIORS: A CROSS-SECTIONAL ONLINE SURVEY AMONG STUDENTS IN BANGLADESH.

[28] Banik R, Rahman M, Sikder MT, Rahman QM, Pranta MUR: Investigating knowledge, attitudes, and practices related to COVID-19 outbreak among Bangladeshi young adults: A web-based cross-sectional analysis. 2020.

[29] Mohsen F, Bakkar B, Armashi H, Aldaher N: A Crisis within a Crisis: COVID-19 Knowledge and Awareness among the Syrian Population—A National Survey Assessment. 2020. 10.1136/bmjopen-2020-043305 PMC8061566 33879483

[30] Zhong et al: Knowledge, attitudes, and practices towards COVID-19 among Chinese residents during the rapid rise period of the COVID-19 outbreak: a quick online cross-sectional survey. *International journal of biological sciences* 2020, 16(10):1745 10.7150/ijbs.45221 32226294 PMC7098034

[31] Haftom et al: Knowledge, Attitudes, and Practices Towards COVID-19 Pandemic Among Quarantined Adults in Tigrai Region, Ethiopia. *Infection and Drug Resistance* 2020, 13:3727 10.2147/IDR.S275744 33116693 PMC7585797

[32] Mya et al: Awareness, perceived risk and protective behaviours of Myanmar adults on COVID-19. *International Journal Of Community Medicine And Public Health* 2020, 7(5):1627.

[33] Lee M, You M: Psychological and Behavioral Responses in South Korea During the Early Stages of Coronavirus Disease 2019 (COVID-19). *International journal of environmental research and public health* 2020, 17(9). 10.3390/ijerph17092977 PMC7246607 32344809

[34] McFadden SM, Malik AA, Aguolu OG, Willebrand KS, Omer SB: Perceptions of the adult US population regarding the novel coronavirus outbreak. *PloS one* 2020, 15(4):e0231808 10.1371/journal.pone.0231808 32302370 PMC7164638

[35] Dryhurst et al: Risk perceptions of COVID-19 around the world. *Journal of Risk Research* 2020:1–13.

[36] Singh A, Ahuja R: Knowledge, Attitude, and Practice of General Public Towards COVID-19 in India: An Online Cross-Sectional Study.

[37] Abdelhafiz et al: Knowledge, Perceptions, and Attitude of Egyptians Towards the Novel Coronavirus Disease (COVID-19). *Journal of community health* 2020:1–10. 10.1007/s10900-019-00710-0 32318986 PMC7173684

[38] Chan et al: Sociodemographic Predictors of Health Risk Perception, Attitude and Behavior Practices Associated with Health-Emergency Disaster Risk Management for Biological Hazards: The Case of COVID-19 Pandemic in Hong Kong, SAR China. *International journal of environmental research and public health* 2020, 17(11). 10.3390/ijerph17113869 32485979 PMC7312582

[39] Lobie TA, Tesfaye D, Abrham A: A narrative synthesis on COVID-19 risks and concerns in developing countries: The case of Ethiopia. *Journal of Public Health and Epidemiology* 2020, 12(2):86–97.

[40] Erhabor O, Erhabor T, Adias T, Okara G, Retsky M: Zero tolerance for complacency by government of West African countries in the face of the COVID-19 pandemic. *Human Antibodies* 2020(Preprint):1–14. 10.3233/HAB-190387 32417768 PMC8150474

[41] Haque et al: Knowledge, attitude and practices (KAP) towards COVID-19 and assessment of risks of infection by SARS-CoV-2 among the Bangladeshi population: An online cross sectional survey. 2020.

[42] Afzal et al: Community-based assessment of knowledge, attitude, practices and risk factors regarding COVID-19 among Pakistanis residents during a recent outbreak: a cross-sectional survey. *Journal of Community Health* 2020:1–11. 10.1007/s10900-019-00710-0 32661860 PMC7356130

[43] Sadiq et al: How COVID-19 is Changing Behaviors of Population-A Study from Punjab? *Biomedica* 2020, 36:239–245.

[44] Qeadan et al: What Protective Health Measures Are Americans Taking in Response to COVID-19? Results from the COVID Impact Survey. *International Journal of Environmental Research and Public Health* 2020, 17(17):6295 10.3390/ijerph17176295 32872439 PMC7503253

[45] Wolf et al: Awareness, attitudes, and actions related to COVID-19 among adults with chronic conditions at the onset of the US outbreak: a cross-sectional survey. *Annals of internal medicine* 2020. 10.7326/M20-1239 PMC7151355 32271861

