# Supplementary material for: Predictors of adherence to COVID-19 prevention measure among communities in North Shoa Zone, Ethiopia based on health belief model: A cross-sectional study
Source: PLoS One. 2021 Jan 22;16(1):e0246006. doi: 10.1371/journal.pone.0246006 (PMC7822535; doi:10.1371/journal.pone.0246006)
Supplement: S1 Annex — (DOCX) [file pone.0246006.s001.docx]

# Annex

## COVID-19 SURVEY QUESTIONNAIRE

Dear Sir/Madam,

Debre Berhan University, College of Health Science, is conducting research on Predictors of adherence to COVID-19 prevention measure among communities in North Shoa Zone, Ethiopia based on health belief model: The finding of the study will helpful to enhance the ongoing public education activities to stop the spread of COVID-19 virus in Ethiopia. Therefore, we kindly request your honest response to this online survey questionnaire. Indeed, your participation is voluntary. But, we highly value your contribution at this critical time. The survey may take about 40 minutes to fill out. Your responses will be completely anonymous. If you have any questions regarding this research, please contact the investigator (Akine Eshete_ 0913460896, Behailu Tariku_0911071777).

Socio demographic characteristics of participants, North Shoa zone

| 00 | Name of Data collector | ________________________ |
| --- | --- | --- |
| 00 | Code of questionnaire | ____________ |
| 101 | Age of the participants | ____________ Years. |
| 102 | Sex of the participants | 1. Male 2. Female |
| 103 | Marital status | 1. Single(unmarried ) 2. Married 3. Separated 4. Divorced 5. Widowed |
| 104 | Religion of participants | 1. Orthodox 2. Muslim 3. Protestant 4. Other(specify___________) |
| 105 | Ethnicity | 1. Amhara 2. Tigirie 3. Oromo 4. Other (specify __________) |
| 106 | Educational status of participants | 1. Illiterate 2. Read and write 3. Primary school (1-8) 4. Secondary school (9-12) 5. Diploma and Certificate 6. Degree and above |
| 107 | Occupational status | 1. Governmental org 2. Private org. 3. Merchant 4. Farmer 5. House wife’s 6. Daily worker 7. Student 8. Other (specify __________) |
| 108 | Place of residence | 1. Urban 2. Rural |
| 109 | Average monthly income | ___________ ETB |
| 110 | Do you have a chronic illness? | 1. Yes 2. No 3. I do not know |
| 111 | Are you a medical professional? | 1. Yes 2. No |

Health Belief model construct questionnaire887

| S. No | Items | 1 (SD) | 2 (D) | 3 (N) | 4 (A) | 5 (SA) |
| --- | --- | --- | --- | --- | --- | --- |
|  | **Perceived Susceptibility** |  |  |  |  |  |
| 301 | I think that I am susceptible to COVID-19 |  |  |  |  |  |
| 302 | I think that only old person are susceptible to COVID-19 |  |  |  |  |  |
| 303 | I think that only person living Urban are susceptible to COVID-19 |  |  |  |  |  |
| 304 | I think that any sex groups are susceptible to COVID-19 |  |  |  |  |  |
| 305 | If I am religious person COVID-19 did not affect me. |  |  |  |  |  |
| 306 | I don't care about this disease and do my daily activities like before |  |  |  |  |  |
| 307 | I think that catching or not catching the COVID-19 is out of my control |  |  |  |  |  |
|  | **Perceived severity** |  |  |  |  |  |
| 308 | I think COVID-19 is a serious disease, if it is not prevented. |  |  |  |  |  |
| 309 | If I had COVID-19, I would be worried and depressed |  |  |  |  |  |
| 310 | COVID-19 is a serious health problem |  |  |  |  |  |
| 311 | I think that COVID-19 is a deadly disease |  |  |  |  |  |
| 312 | I think that COVID-19 is highly infectious |  |  |  |  |  |
|  | **Perceived Benefits** | **SD** | **D** | **N** | **A** | **SA** |
| **313** | I believe that recommended hand washing is helpful for me, to prevent myself from COVID-19 |  |  |  |  |  |
| 314 | I believe that recommended social distancing is helpful for me, to prevent myself from COVID-19 |  |  |  |  |  |
| 315 | I believe that staying at home is helpful for me, to prevent myself from COVID-19 |  |  |  |  |  |
| 316 | I believe that avoiding from overcrowding place is helpful for me, to prevent myself from COVID-19 |  |  |  |  |  |
| 317 | I believe that praying out of religion place/home is helpful for me, to prevent myself from COVID-19 |  |  |  |  |  |
| 318 | I believe that the government restriction is helpful for me, to prevent myself from COVID-19 |  |  |  |  |  |
| 319 | I believe that the information regarding COVID-19 is helpful for me, to prevent myself from COVID-19 |  |  |  |  |  |
| 320 | I believe that, when I stop shaking people’s hand is helpful for me, to prevent myself from COVID-19 |  |  |  |  |  |
| 321 | I believe that, when I respect the rules and regulations set by MoH& government is helpful for me, to prevent myself from COVID-19 |  |  |  |  |  |
| 322 | When I wash my hands after coughing or sneezing, I am doing something to care for myself and my families. |  |  |  |  |  |
|  | **Risk of having COVID-19** |  |  |  |  |  |
| 323 | If I do not have symptoms, I do not need a COVID-19 test |  |  |  |  |  |
| 324 | If I do not have symptoms, I do not need a social distancing |  |  |  |  |  |
| 325 | If I have not had contact with infected person, I do not need a to have hand washing |  |  |  |  |  |
| 326 | I am drinking more alcohol to prevent COVID-19 |  |  |  |  |  |
| 327 | If one of my family member/ friend infected with covid-19 self-isolation is not needed |  |  |  |  |  |
| 328 | If I have COVID-19, I am not worry for other |  |  |  |  |  |
|  | **Perceived Barriers** | SD | D | N | A | SA |
| 329 | I have no knowledge how to prevent COVID-19 |  |  |  |  |  |
| 330 | It is difficult to find water and soap at any place |  |  |  |  |  |
| 333 | I do not wash my hands because it take long time |  |  |  |  |  |
| 332 | I do not wear face mask because the mask is scarce in the market. |  |  |  |  |  |
| 333 | I don’t use alcohol based sanitizers because it’s scarcity in the market. |  |  |  |  |  |
| 334 | It is difficult not to touch hands, mouth, nose and eyes |  |  |  |  |  |
| 335 | Staying at home to prevent the disease is difficult |  |  |  |  |  |
| 336 | Washing hands repeatedly costs much and I and my families will not afford |  |  |  |  |  |
| 337 | It is hard to remember washing hands repeatedly. |  |  |  |  |  |
| 338 | I can’t stop shaking hands because my relation with people become affected |  |  |  |  |  |
| 339 | I can’t stop going to religious places because my God protect me from any diseases. |  |  |  |  |  |
| 340 | I can’t keep physical distancing because it is difficult |  |  |  |  |  |
|  | **Cues to action** | **SD** | **D** | **N** | **A** | **SA** |
| 341 | I have been looking for TV and radio to get helpful information about the disease |  |  |  |  |  |
| 342 | I am looking for helpful information from peers about the disease. |  |  |  |  |  |
| 343 | Talking with health professionals about the disease is helpful |  |  |  |  |  |
|  | **Self‐efficacy** | **SD** | **D** | **N** | **A** | **SA** |
| 344 | I have ability to follow every preventive instructions against the disease |  |  |  |  |  |
|  | **Likelihood of taking recommended action** | **Never** | **Rarely** | **Sometimes** | **Often** | **Always** |
| 345 | I have practiced recommend hand washing practice for at least 20 seconds |  |  |  |  |  |
| 346 | I have practiced avoiding touching eyes, nose, and mouth with unwashed hands |  |  |  |  |  |
| 347 | I have practice use of disinfectants to clean hands when soap and water was not available for washing hands |  |  |  |  |  |
| 348 | I have been staying home when I was sick or when I had a cold |  |  |  |  |  |
| 349 | I have been practicing covering my mouth and nose when I cough or sneeze |  |  |  |  |  |
| 351 | I have been practicing physical distancing at least 2-metter away from others |  |  |  |  |  |
| 352 | I have been practicing self-isolation when I have fever, cough and headache |  |  |  |  |  |
| 353 | I have been practicing disinfecting surfaces that belongs to me. |  |  |  |  |  |
| 354 | I have been wearing a face mask when I go to crowding area |  |  |  |  |  |
| 355 | I am using antibiotics for prophylaxis |  |  |  |  |  |
| 356 | I am using Herbal supplements as prophylaxis |  |  |  |  |  |
| 357 | I am using homeopathic remedies as prophylaxis |  |  |  |  |  |
| 358 | I am disinfecting my mobile phone with alcohol based sanitizer |  |  |  |  |  |
| 359 | I am eating Garlic, Ginger, Lemon, Feto, |  |  |  |  |  |

Knowledge of COVID-19 and source of the information

| 401 | Which of the following sources of information you use to know about the novel coronavirus?  multiple answer is possible | 1. Television 2. Radio 3. Health care professional 4. family and/or friends 5. Social media (e.g. Facebook, Twitter, YouTube, WhatsApp) 6. Official, government press releases 7. Religious announcement 8. Other |
| --- | --- | --- |
| 402 | Which of the following sources of information you trust about the novel coronavirus? multiple answer is possible | 1. Television 2. Radio 3. Health care professional 4. family and/or friends 5. Social media (e.g. Facebook, Twitter, YouTube, WhatsApp) 6. Official, government press releases 7. Religious announcement 8. Other |
| 403 | Is headache a main symptom of this disease? | 1. YES 2. NO 3. I don’t know |
| 404 | Is runny nose a main symptom of this disease? | 1. YES 2. NO 3. I don’t know |
| 405 | Is fever a main symptom of this disease? | 1. YES 2. NO 3. I don’t know |
| 406 | Is dry cough a main symptom of this disease? | 1. YES 2. NO 3. I don’t know |
| 407 | Is shortness of breath a main symptom of this disease? | 1. YES 2. NO 3. I don’t know |
| 408 | Is body and muscle pain the main symptoms of this disease? | 1. YES 2. NO 3. I don’t know |
| 409 | Are digestive problems (diarrhea and nausea) the main symptoms of this disease? | 1. YES 2. NO 3. I don’t know |
| 410 | Are People who have contact with someone infected with the COVID-19 virus should be immediately isolated in a proper place? | 1. YES 2. NO 3. I don’t know |
| 411 | A person with COVID-19 cannot infect the virus to others when sign and symptom is not present | 1. YES 2. NO 3. I don’t know |
| 412 | It is not necessary for children and young adult to take measures to prevent COVID-19 | 1. YES 2. NO 3. I don’t know |
| 413 | Does COVID-19 virus spreads from infected person to uninfected person Via respiratory droplets of infected individuals during sneezing or coughing | 1. YES 2. NO 3. I don’t know |
| 414 | Does COVID-19 virus spreads from infected person to uninfected person Via respiratory droplets of infected individuals during speaking | 1. YES 2. NO 3. I don’t know |
| 415 | Does COVID-19 virus spreads from infected person to uninfected person Via direct contact with contaminated hands, fomite, surfaces, etc | 1. YES 2. NO 3. I don’t know |
| 416 | Does COVID-19 virus spreads from infected person to uninfected person Via airborne (droplet nuclei of the virus or dust particles containing virus can remain in air and inhaled) | 1. YES 2. NO 3. I don’t know |

Thank you very much for your information and time!
